# Dermatologic manifestations in paediatric neurofibromatosis type 2: a cross sectional descriptive multicentric study

**DOI:** 10.1186/s13023-022-02379-6

**Published:** 2022-06-21

**Authors:** S. Legoupil, D. Bessis, F. Picard, S. Mallet, J. Mazereeuw, A. Phan, D. Dupin-Deguine, M. Kalamarides, Research Group of the French Society of Paediatric Dermatology, C. Chiaverini

**Affiliations:** 1 Department of Dermatology, CRMRP-Sud, ARCHET 2 Hospital, CHU de Nice, 151 route st Antoine de Ginestière, 06200 Nice, France; 2 Department of Pediatrics, CHU de Nice, Fondation Lenval, Nice, France; 3 Department of Dermatology, CHU de Montpellier, Montpellier, France; 4 Department of Dermatology, CHU de Bordeaux, Bordeaux, France; 5 Department of Dermatology, APHM, Timone Enfant de Marseille, Marseille, France; 6 Department of Dermatology, CHU de Toulouse, Toulouse, France; 7 Department of Pediatric Dermatology, Hospices Civils de Lyon, Lyon, France; 8 Department of ENT, CHU de Toulouse, Toulouse, France; 9 Department of Neurosurgery, APHP, Pitié-Salpêtrière, Paris, France; 10 CNR-NF2, APHP, Pitié-Salpêtrière, Paris, France

**Keywords:** Neurofibromatosis 2, Dermatology, Paediatric

## Abstract

**Background:**

Neurofibromatosis type 2 (NF2) is characterized by bilateral vestibular schwannoma (VS) more often in adults but a severe paediatric form with multiple neurological tumours is also described. In this population, a early diagnosis is important to prevent the onset of neurological complications but is difficult, particularly without a familial history. Cutaneous manifestations, which may precede VS or neurological tumours by several years, may contribute to an early diagnosis, but specific studies are lacking. The objective of this study was to characterize cutaneous manifestations of NF2 in a paediatric population.

**Results:**

This observational, descriptive and multicentric study was conducted from April 2019 to April 2020 in seven academic French hospitals. We included patients ≤ 18 years old who fulfilled the Manchester diagnostic criteria or had a pathogenic mutation identified in the *NF2* gene. All patients underwent a dermatological examination guided by a standardized questionnaire. 21 children were included, of whom 20 had at least one skin tumour (mean number 5 ± 4.6 [range 0–15]), which led to a diagnosis in four cases. In the other 17 cases, the diagnosis of NF2 was based on neurosensory complications (n = 10), family screening (n = 4) or ocular signs (n = 3). Before the NF2 diagnosis, 15 children had at least one “undiagnosed” cutaneous tumour that did not lead to a specific management. Patients’ dermatological examination also revealed < 6 non specific *café au lait* macules (n = 15), hypopigmented macules (n = 12) with more than 3 lesions in 4 cases, and purple reticulated macules of the trunk (n = 4).

**Conclusion:**

Dermatological lesions are frequent and early in children with NF2 but rarely lead to the diagnosis. Cutaneous schwannomas are the most frequent but are often underdiagnosed. *Café au lait* macules are frequent, but atypical and mostly in small numbers. Multiple hypopigmented macules seem suggestive although inconsistent. The sensitivity of reticulated capillary malformation-like lesions remains to be assessed by further studies.

**Supplementary Information:**

The online version contains supplementary material available at 10.1186/s13023-022-02379-6.

## Introduction

Neurofibromatosis type 2 (NF2), a rare genodermatosis with an incidence of 1/25–33,000 births and a prevalence of 1/60,000 [1–3], is linked to an autosomal dominant mutation of the *NF2* gene, located on chromosome 22. The average age at diagnosis, based on Manchester criteria or the identification of a pathogenic mutation in *NF2*, is 17 to 22 years old [4]. In up to 20% of cases, especially sporadic ones, no pathogenic mutation in *NF2* is identified [5]. Clinically, NF2 is characterized by the occurrence of bilateral or unilateral vestibular schwannomas (VS), associated with benign central nervous system (CNS) tumours localized in brain, spinal or cranial nerves. The main other presenting features are ophthalmic and dermatological [2, 6–9]. Two major phenotypes have been described: mild (Gardner type) with bilateral VS becoming symptomatic in young adults, and severe (Wishart type), with multiple CNS tumours and cutaneous and ophthalmological involvement, starting from childhood [10–12].

In this paediatric population, an early diagnosis is important to prevent the onset of neurological complications but is difficult, particularly without a familial history. Cutaneous or ophthalmological manifestations, which may precede VS or CNS tumours by several years, may contribute to an early diagnosis [11–13]. However, apart from cutaneous schwannomas, the literature contains few descriptions of dermatological manifestations [8, 11, 13–17].The aim of this study was to characterize the dermatological manifestations of NF2 in a paediatric population.

## Materials and methods

This observational, cross sectional and multicentric study was conducted from April 2019 to April 2020 in seven academic French hospitals. Patients of both sexes, ≤ 18 years old, fulfilling the Manchester diagnostic criteria or with a pathogenic mutation identified in the *NF2* gene, were included after oral consent. Recruitment was by dermatologists from the Research Group of the French Society of Paediatric Dermatology, the French reference centre for NF2 and the French association of patients with neurofibromatosis.

All patients had a dermatological examination, with Wood’s lamp examination and photography, guided by a standardized questionnaire with an atlas of the various types of schwannomas according to Mautner et al. [8] and ophthalmologic, neurologic and otolaryngeal (ENT) features were collected. According to the literature, the clinical phenotype was considered severe for children with non-VS neurological-related symptoms and mild for the others [10–12]. *NF2* mutations were stratified by using the previously published and validated UK genetic severity score [18]. Briefly, group 1A/1B represents no *NF2* pathogenic variant detected in blood; groups 2A and 2B represent a mild or moderate, respectively, constitutional or mosaic *NF2* pathogenic variant detected in blood; and group 3 represents a constitutional truncating pathogenic variant within exons 2–13.

The study was considered non-interventional by our ethics committee and was registered at ClinicalTrials.gov (NCT03893643). All patients and/or their caregivers gave their written consent to use their pictures in publications.

## Results

### Patients

We included 21 children (mean age 13 ± 4.2 years [range 2–18]; sex ratio 11 boys/10 girls) (Table 1). NF2 was sporadic in 16 cases and familial in five. The phenotype was severe in 18 children and moderate in 2; one child was asymptomatic. NF2 was diagnosed because of neurological complications due to tumour (n = 10; median age 9 ± 3.2 years) in patients without a family history, family screening (n = 4; median age 11 ± 1.2 years), with cutaneous signs (n = 4, median age at diagnosis 12 ± 5.4 years; median age at first signs 7 ± 4.9 years) or ocular signs (n = 3; median age at diagnosis 4 ± 6.4 years; median age at first signs 0.5 ± 0.7 years). Detailed clinical and genetic data of each patient are available on Additional file 1: Table S1.

**Table 1:** Characteristics of the population, genetic data, and dermatologic, ophthalmologic, neurologic and ENT features. yo: years old; M: male; F: female; CALMs: *café-au-lait* macules; HPMs: hypopigmented macules

	Patients
Sex	11 M/10F
Median age at time of study	13 ± 4,2 yo [range 2–18]
Median age at first sign	8 ± 4,3 yo [range 0–15]
Symptoms leading to diagnosis / Median age at diagnosis	Neurological complications (n = 10)/9 ± 3,2 yo [range 6–15] Family screening (n = 4)/11 ± 1,2 yo [range 8–11] Cutaneous signs (n = 4)/12 ± 5,4 yo [range 3–15] Ocular signs (n = 3)/4 ± 6,4 yo [range 2–14]
Phenotype	severe = 18/moderate = 2/asymptomatic = 1
Family history	Negative 16/Positive 5
Dermatological features	
*Median Number of skin tumours*/*median age of onset*	5 ± 4,5 [range 0–15]/7 ± 5,4 yo [range 0–16]
Type A or AH	2 ± 3,9 [range 0–15]/(n = 15)
Type B	0,5 ± 1,4 [range 0–5]/(n = 11)
Type C	0 ± 2,4 [range 0–8]/(n = 10)
Type D	0 ± 0,2 [range 0–1]/(n = 2)
*CALMs*	1 ± 1,6 [range 0–5]/(n = 15)
*HPMs*	1 ± 2,1 [range 0–7]/(n = 12)
*Purple lesion*	0 ± 0,5 [range 0–2]/(n = 4)
Ophtalmological features	
Retinal lesions	0 ± 0,37 yo [range 0–1]/(n = 9)
Posterior cataracts	6 ± 7 yo [range 4–17]/(n = 4)
Strabismus	1 ± 4,5 yo [range 0–10]/(n = 8)
Optic meningioma	total (n = 4)/unilateral (n = 3)/bilateral (n = 1)
Neurological features	
*Median number of tumour*	7 ± 4,4 [range 0- > 10]/(n = 18)
*Tumour location*	
Brain	n = 14
Spine	n = 14
Cranial nerves (except N.VIII)	n = 7
*Histological type*	
Schwannoma	2 ± 1,7 [range 0- > 10]/(n = 14)
Meningioma	2 ± 1,5 [range 0–4]/(n = 12)
Ependymoma	0 ± 3,6 [range 0–12]/(n = 5)
Astrocytoma	(n = 0)
ENTs features	
Vestibular schwannoma	total (n = 19)/unilateral (n = 2)/bilateral (n = 17)

### Ocular features

Seventeen children had a specific ophthalmological involvement, with retinal lesions (n = 9), strabismus (n = 7) and posterior cataracts (n = 4). Retinal lesions included retinal hamartomas (n = 7, six congenital and one diagnosed at age 1 year), and congenital epiretinal membranes (n = 2). Strabismus was congenital in four children and appeared at age 11 months and 8 and 10 years for the three others. Cataracts were diagnosed at a median age of 6 ± 7 years [range 4–17].

Eight children had a visual deficit because of isolated or associated brain tumours (n = 2), bilateral or unilateral optic nerve meningioma (n = 4), large retinal hamartoma (n = 1), VI nerve palsy associated with myopia and ipsilateral divergent strabismus (n = 1) or amblyopia (n = 5) secondary to retinal lesions and/or posterior cataract.

Ophthalmologic anomalies led to an NF2 diagnosis for three children: one at age 2 years, with two retinal hamartomas diagnosed at age 1 year; one with retinal detachment at age 14 years resulting from congenital retinal hamartoma; and one because of posterior cataract at age 4 years.

### Neurosensory features

Seventeen children had CNS tumours. Tumours were localized in the brain (n = 14), spine (n = 14) and/or cranial nerves (n = 7). Eight children had multiple cauda equine schwannomas. Ten children had more than 10 tumours. The different histological types were schwannomas (63%), meningiomas (20%) and ependymomas (17%). Complications related to these tumours led to an NF2 diagnosis in seven patients, with headache (n = 2; median age 6.5 years [range 6–7]), cervicalgia (n = 2; median age 11.5 years [range 9–14]), brutal motor deficit (n = 1; age 9 years), intracranial hypertension (n = 1) or genito-sphincter disorders (n = 1; age 14 years). Epilepsy (n = 3; median age 11 ± 2.1 years [10–14]) and attention deficit disorder with hyperactivity (n = 1; age 8 years) were also noted.

### ENT features

Most children (19/21) had bilateral (n = 16) or unilateral (n = 3) VS (median age at diagnosis 10.5 ± 3.4 years [range 3–15]): 12 were already symptomatic (median age at first symptoms 12 ± 3.2 years [range 6–15]). Symptoms included hearing loss (n = 7; median age 12 ± 3.4 years [6–15]), which led to an NF2 diagnosis in three cases, peripheral facial paralysis (n = 5; median age 9 ± 3.9 years [range 6–15]) or vestibular damage (n = 3; median age 11 ± 4.5 years [range 6–15]). Only a 14-year-old patient with an asymptomatic NF2 and a 2-year-old child did not have VS.

### Dermatological features

**Fig. 1:**
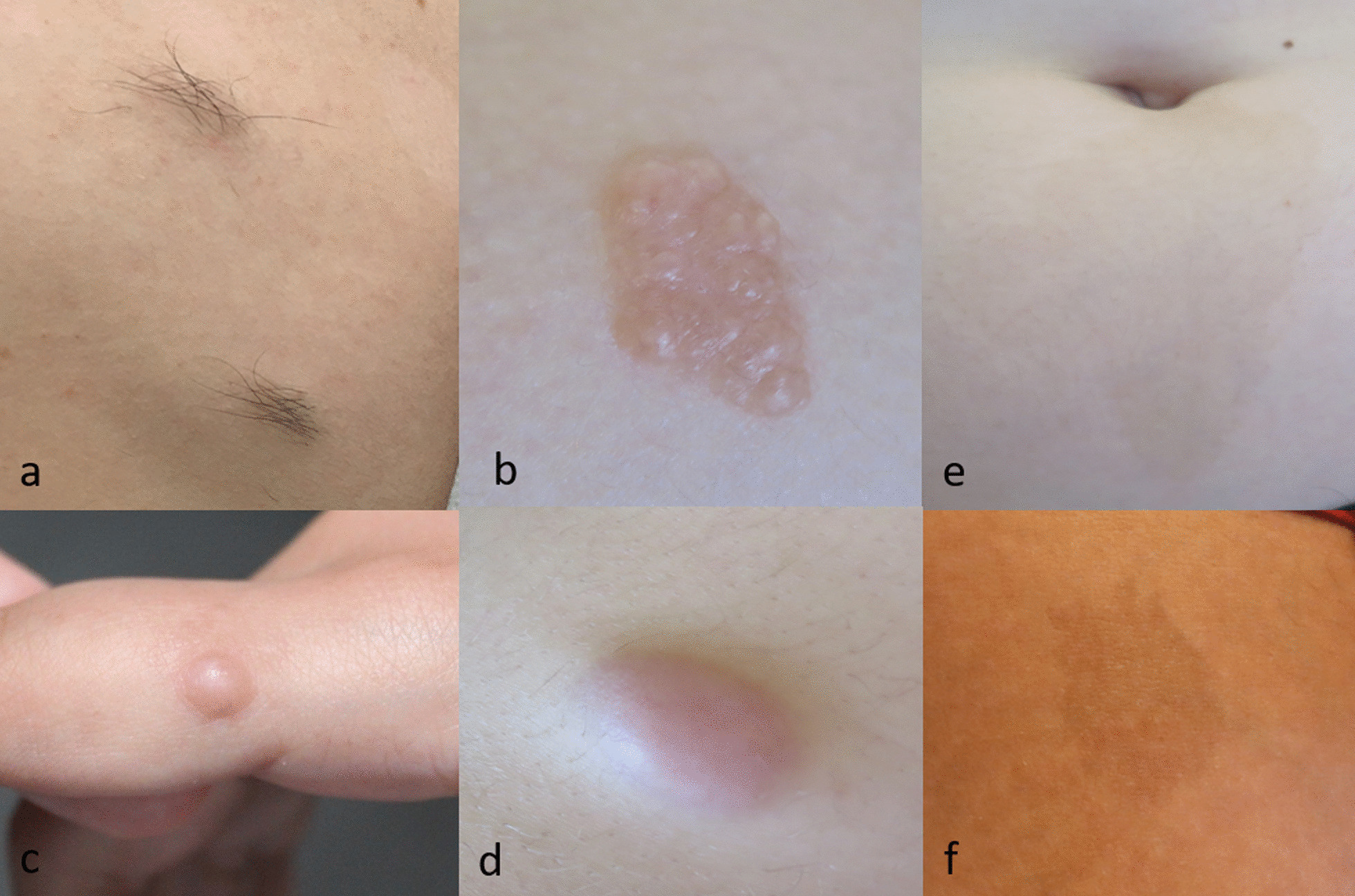
Classical dermatological manifestations in patients with NF2. Type A/AH tumours: pigmented plaques, rough to the touch, with (**a**) or without (**b**) hypertrichosis. Type B tumours: subcutaneous spherical nodulary tumours on peripheral nerves (**c**). Type C tumours: pink nodular epicutaneous tumours (**d**). Atypical *café-au-lait* macules with, irregular margins and ragged borders (**e**, **f**)

Almost all children (20/21) had at least one skin tumour, which appeared before age 10 in 14 children and were congenital in four. The median number of tumours was 5 ± 4.6 [range 0–15] per patient, and five children had more than 10 skin tumours. Children with the moderate form had 0 to 3 tumours. Type A or AH tumours were the predominant subtype and were found in 15 children (Fig. 1a, b), type B tumours in 11 (Fig. 1c), type C tumours in 10 (Fig. 1d), and type D in only two. Histology concluded schwannomas (n = 9) or neurofibromas (n = 2).

Five children specifically consulted for a skin tumour, with a clinical diagnosis of lipomas for two patients, neurofibromas in one child and suspicious lesion in the last two cases. Four of these children underwent resection with cytopathological examination leading to the diagnosis of schwannoma (n = 3) or neurofibroma (n = 1) and subsequently NF2 diagnosis at 1, 6, 8 and 13 yo respectively. No correlation between the early onset of skin tumour and the severity of neurological signs was found.

Fifteen children had *café-au-lait* macules (CALMs). The median number of CALMs was 2 ± 1.5 [range 1–5] per patient, sometimes congenital (n = 3). The size was > 15 mm in 50%, borders were mainly ragged, and the colour was homogeneous within the same spot but heterogeneous between the different lesions (Fig. 1e, f). CALMs were mainly localized on the trunk (50%) and limbs (41%) and sometimes the face (9%).

**Fig. 2:**
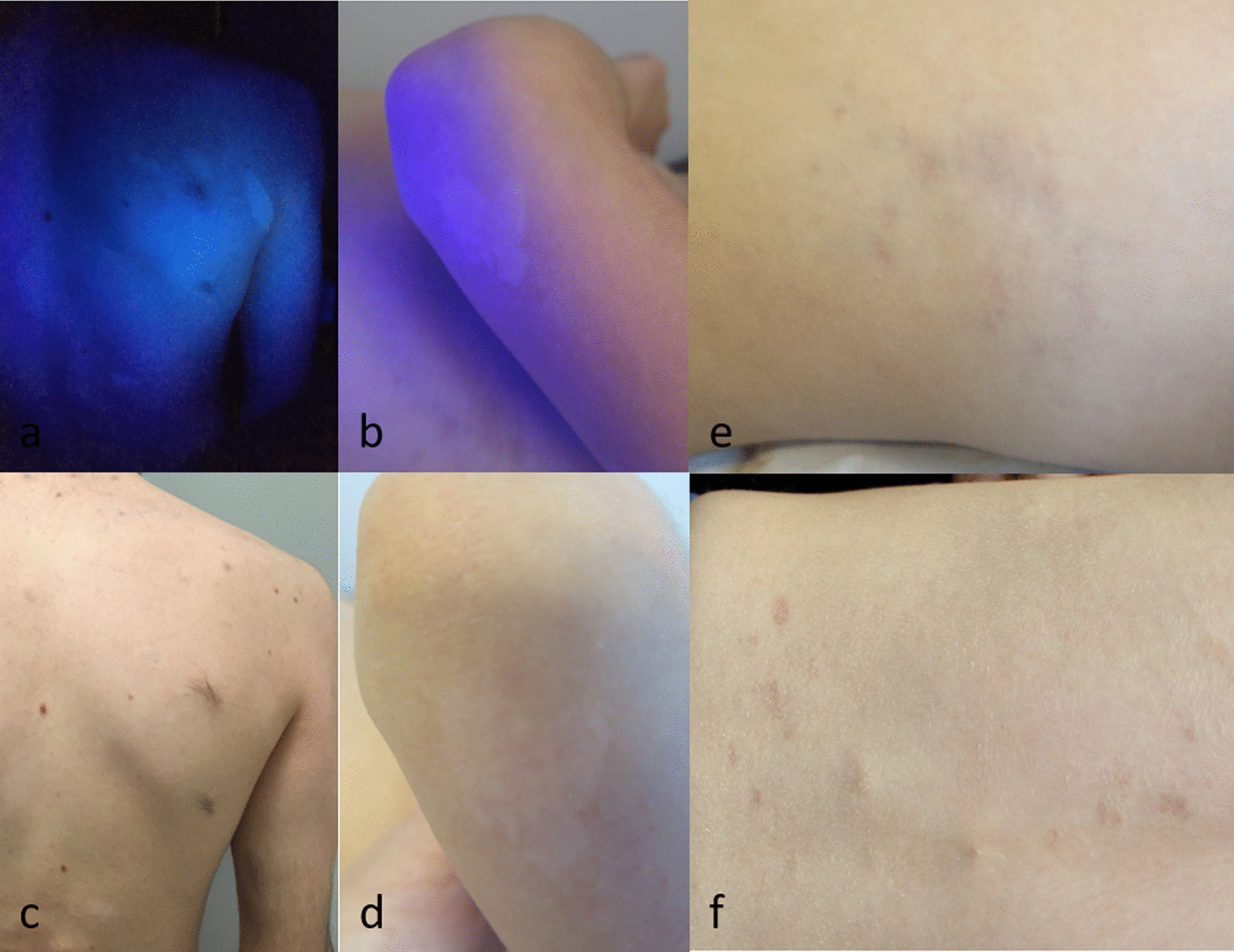
New dermatological manifestations in patients with NF2. Multiple large hypopigmented macules of the trunk in a teenager (**a**), large hypopigmented macule with ragged borders of an elbow in a young girl (**b**), highlighted with Woods’ lamp (**c**, **d**). Heterogeneous, reticulated, congenital purple macule on the trunk in two children (**e**, **f**)

Twelve children showed hypopigmented macules (HPMs), which were congenital (n = 1), appeared at the age of 5 years (n = 1) or at unknown age of onset (n = 10) and were mainly large (88% > 15 mm), with ragged borders. The median number was 3 ± 2.2 [range 1–7] per patient, with at least four spots in four children. The spots could be discrete, only highlighted by Wood’s lamp (Fig. 2a–d).

Finally, four children had a heterogeneous, reticulated, purple macule localized on the trunk or lower limbs, which was congenital in one case (Fig. 2e, f). These lesions were discrete and disappeared at the vitropression, which suggested a vascular origin, but no Doppler ultrasonography/ dermoscopy analysis/skin biopsy was performed.

None of these lesions led to an NF2 diagnosis. No other skin or mucosal lesion was identified.

### Genetic data

Mutation analysis of the *NF2* gene could be performed for 16 children. The other five children or parents refused the genetic test. A pathogenic mutation in the *NF2* gene was found in 14 children (no mutation found in two patients). For the three children with a mild phenotype, the first presented a complete deletion of the *NF2* gene; the second, a truncating pathogenic variant in exon 11 at mosaic state (15%); and the last, a mutation of intron 11 leading to a splicing anomaly. This last child also had a multiple endocrine neoplasia type 2. Children with a severe phenotype had various constitutional mutations (in-frame large deletion, n = 2; nonsense mutation, n = 4; missense mutation, n = 1; splice site mutation, n = 1) and one mosaic (25%) in-frame deletion of a nucleotide. Among the two mosaic cases, one with a diagnosis at age 2 years, presented a moderate form, with only early cutaneous and ocular signs at age 4 years, and the second, with a diagnosis at age 14 years, presented a severe form revealed by serious neurosensory complications revealing multiple cerebral-medullary tumours. The genetic severity score varied from 1A to 3.

## Discussion

This study is the first to systematically and prospectively search for skin anomalies by clinical examination performed by a dermatologist in a paediatric cohort with NF2. Six other studies analysed skin lesions in NF2 patients [2, 7, 8, 11–13]: three were retrospective, three included adults and children, and none included an examination by a dermatologist. Comparisons of results are summarized in Table 2.

**Table 2:** Summary of studies published on skin features in children with neurofibromatosis type 2. ND: not diagnosed; A: adult; C: child; CALMs: *café au lait* macules; HPMs: hypopigmented macules

	Our study	Gugel et al. [13]	Anand et al. [11]	Ruggieri et al. [12]	Mautner et al. [8]	Parry et al. [7]	Evans et al. [2]
Cohorte	21 (C)	70 (C)	32 (C)	22 (C)	88 (A + C)	63 (A + C)	100 (A + C)
Dermatologist examination	Yes	No	No	No	No	No	No
Data collection	Prospective	Retrospective	Retrospective	Prospective	Prospective	Prospective	Retrospective
Median age (years old)	13 [2–18]	11 [1–17]	3,5 [0–15]	12,5 [4–18]	ND	34,6 [7–71]	ND
Sporadic cases	16 (76%)	55 (78%)	32 (100%)	19 (86%)	ND	17 (27%)	ND
Severe phenotype	18 (85%)	ND	15 (47%)	22 (100%)	62 (71%)	38 (60%)	46 (46%)
Skins tumours	20 (95%)	31 (44%)	20 (62,5%)	21 (95%)	52 (59%)	9 (14%)	68 (68%)
Type A/AH	15 (71%)	ND	12 (38%)	19 (86%)	36 (40%)	ND	ND
Type B	11 (52%)	ND	8 (25%)	9 (41%)	42 (47%)	ND	ND
Type C	10 (47%)	ND	ND	4 (18%)	7 (8%)	ND	ND
Type D	2 (9%)	ND	ND	ND	6 (7%)	ND	ND
CALMs	15 (71%)	1 (1,4%)	12 (38%)	8 (36%)	29 (33%)	29 (46%)	43 (43%)
HPMs	12 (57%)	ND	3 (9%)	ND	ND	ND	ND
purple Lesions	4 (19%)	ND	ND	ND	ND	ND	ND

The prevalence of skin tumours in our cohort was high (n = 20, 95%) and comparable to that found by Rugierri et al. (95%) [12], in the only prospective study of children with severe NF2, but higher than the five other studies (14–68%). These differences could be explained by these studies also including adults and/or their retrospective design and/or including patients with milder phenotype. We found, as others did, type A or AH tumours the most represented, before types B, C and D.

Multiple CALMs, a major clinical diagnostic criterion of neurofibromatosis type 1, have also been reported in NF2, with which it was initially confused. We found CALMs in 15 (71%) children, a higher prevalence than in other studies (1.4–46%), probably because of the skin examination with a Wood’s lamp by a dermatologist. However, none of our children had at least six CALMs, and only four had more than three CALMs, with a median of 2 ± 1.5. Furthermore, analysis of semiological characteristics of CALMs showed that they were atypical [19], with small size, irregular margins and ragged borders, and different colours in the same individual. Because of the high prevalence of CALMs in the general population (18.9–36%) [19], the specificity and sensitivity of this clinical sign in NF2 patients seems low, with CALMs not a cardinal feature of NF2.

We showed a high prevalence (n = 12, 57%) of HPMs in children with NF2. They were mainly large > 15 mm, with irregular margins and ragged borders, and could be missed without careful examination with a Wood’s lamp. Although the age of onset was difficult to assess, HPMs were congenital in one case and noted at age 5 years in second case. Four children had more than three HPMs (range 4–7), but nine children had no lesions. We found three articles in the literature concerning these lesions. Anand et al. [11] reported a prevalence of 9% of “hypopigmented lesions” in a retrospective study including 32 children with NF2. Casado-Verrier et al. [14] and Miyakawa et al. [20] both reported multiple HPMs in a case report of a 7- and 5-year-old child, respectively. No clinical description was available in any of these articles. The five other studies that analysed skin signs in NF2 patients did not report such lesions. Isolated HPMs are a common feature in a paediatric population, with a prevalence estimated at 4.7% [21], but multiple (more than three) HPMs could be an inconstant but specific and early sign of NF2.

Finally, four children in our series had flat purple lesions of the trunk not previously described, whose sensitivity in NF2 remains to be determined. These lesions were large (> 5 cm), asymptomatic and discrete. Unfortunately, no Doppler ultrasonography/dermoscopy/skin biopsy was performed to confirm the vascular origin of these lesions, which is a limitation of this study. There is no other case reported in the literature.

Despite their precocity and high prevalence, dermatological lesions, that is, cutaneous tumours, led to the NF2 diagnosis in only four cases after excision and discovery of a schwannoma (n = 3) or neurofibroma (n = 1). Therefore, before the NF2 diagnosis, 15 (71%) children had at least one “undiagnosed” cutaneous tumour (but sometimes more than five) that did not lead to a specific management. We found similar results with ophthalmologic symptoms with only 3 diagnoses despite 17 patients with ocular lesions [22]. Indeed, in our series, NF2 was diagnosed in 10 (47%) patients because of neurological complications (including hearing loss), similarly to Anand et al. (56%) [11] and Gugel el al. (53%) [13] studies. Interestingly, before this diagnosis, seven of these children had a combination of evocative skin and ophthalmic lesions but none met the Manchester diagnostic criteria, which not include the most frequent ophthalmic feature found in our series ie congenital retinal lesions.

These data led to several considerations: 1) childhood onset of NF2 is a severity factor, and early diagnosis is mandatory to improve the outcome of patients; 2) the Manchester diagnostic criteria are not adapted to paediatric populations; 3) paediatricians and dermatologists must be aware to not leave skin lesions undiagnosed in children, especially if they are multiple, if their number increases over time and/or if they are associated with congenital ophthalmic lesions; 4) the presence of suggestive early ocular signs, particularly retinal lesions, must encourage the ophthalmologist to refer their patient to other specialists, looking for further symptoms suggestive of NF2; and 5) clear guidelines for NF2 screening in case of suggestive neurological or non-neurological symptoms are mandatory for this population. This would require compiling a list of ophthalmological and dermatological signs suggestive of the diagnosis of NF2 in childhood as proposed by Anand et al. [11]. In this way, further studies to assess the sensitivity and specificity of HPMs and purples macules in larger cohorts of NF2 patients would be of interest.

Finally, regarding the molecular analysis of patients, an NF2 pathogenic variant was found in 14/16 (87.5%) children, which is consistent with the literature. Recently a genetic severity score was proposed by Halliday et al. [18], with three groups: 1A/1B: no NF2 pathogenic variant in blood; 2A/2B: mild/moderate NF2 constitutional or mosaic pathogenic variant in blood; and 3: constitutional truncating exon 2–13 pathogenic variant. Patients in group 3 may show disease earlier, with greater tumour load, and have poorer visual outcomes and require more interventions [18, 23]. In our series, a severe phenotype was detected in two children with no pathogenic variant detected in blood and without molecular analysis of tumours; one patient with an in-frame deletion of a nucleotide in mosaic state; and one patient with a missense mutation (score 1A to 2A), which suggests that the phenotype–genotype correlation is difficult to clearly establish in the paediatric population.

## Conclusions

Dermatological and ophthalmic lesions are frequent and early in children with sporadic severe NF2 but rarely lead to the diagnosis because of lack of clear guidelines. Cutaneous tumours, and in particular schwannomas, are the most frequent dermatological lesions but are often underdiagnosed. CALMs are frequent, but atypical, and mostly in small numbers. Multiple HPM lesions seem suggestive although inconsistent. The sensitivity of reticulated capillary malformation-like lesions remains to be assessed by further studies.

## Supplementary Information

**Additional file 1**: Characteristics of the population, genetic data, and dermatological, ophthalmological, neurological and otolaringeal (ENT) features. yo: years-old; d: deceased; ND: not done; M: male; F: female; CALMs : Café-Au-Lait Macules; HPMs : Hypopigmented Macules.

## Data Availability

Yes.
