# Supplementary material for: Dermatologic manifestations in paediatric neurofibromatosis type 2: a cross sectional descriptive multicentric study
Source: Orphanet J Rare Dis. 2022 Jun 21;17:242. doi: 10.1186/s13023-022-02379-6 (PMC9210598; doi:10.1186/s13023-022-02379-6)
Supplement: Supplementary file 1 [file 13023_2022_2379_MOESM1_ESM.docx]

Patient	1	2	3	4	5	6	7	8	9	10	11	12	13	14	15	16	17	18	19	20	21
**Sex**	F	M	M	F	F	M	F	M	F	M	M	M	F	M	F	F	M	F	F	M	M
**Age (yo) at firsts symptoms / at diagnosis / at time of study**	2 / 4	3 / 3	4 / 8	6 / 14	7 / 10	7 / 10	8 / 9	9 / 9	ND / 10	9 / 11	10 / 13	11 / 13	11 / 13	ND / 14	11 / 14	12 / 17	14 / 15	14 / 17	14 / 17	15 / 17	15 / 18
**Phenotype**	Mild	severe	severe	severe	severe	severe	severe	severe	severe	severe	severe	mild	severe	severe	mild	severe	severe	severe	severe	severe	severe
**Family history**	No	No	No	No	No	No	Yes	No	Yes	No	No	Yes	No	Yes	Yes	No	No	No	No	No	No
**Manchester Criteria fulfilled**	No	Yes	Yes	Yes	Yes	Yes	Yes	Yes	Yes	Yes	Yes	Yes	Yes	Yes	No	Yes	Yes	Yes	Yes	Yes	Yes
**Mutation Nucleotide change / protein change**	c.1183delG / p.(Ala395Glnfs*31) **Mosaicism 15%**	Deletion of exon 10-13 c.886-?81446+?del / undefined	c.1-?_114 ?del / undefined	Mutation in exon 2 c.113delG / p.Asp45fs*	ND	Mutation in exon 2 c.169C>T / p.Arg57fs*	No mutation Found	exon 2 deletion	ND	No mutation Found	Mutation in Intron 4 c.447+2T > C / undefined	complete deletion of the NF2 gene	c.114G>C / p.Glu38Asp	ND	c.1123-3C>G / p.(Met375fs*) + MEN2	ND	ND	Mutation in Exon 3 c.357_359delCTT / p.Phe119del **Mosaicism 25%**	Mutation in Exon 6 c.586C>T / p.Arg196fs*	Mutation in exon 1 c.84delC / p.Met29Trpfs*11	Mutation in Exon 12 c.1137_1138delGA / undefined
**Genetic severity score** Halliday et al. (18)	2B	2B	2A	2B	ND	3	1A	2B	ND	1A	2A/B	2A	2A	ND	3	ND	ND	2A	3	2A	3
**symptoms leading to diagnosis / Age of onset**	Ophtalmological signs (retinal hamartoma) / 1 yo	Cutaneous signs (schwannomas) / 1 yo	Ophtalmological signs (cataract) / < 4 yo	Neurosensory complications (headache + reduced visual acuity) / 6 yo	Neurosensory complications (hearing loss) / 7 yo	Neurosensory complications (headache) / 7 yo	Family screening / 8 yo	Neurosensory complications (torticollis + cervicalgia) / 9 yo	Neurosensory complications (intracranial hypertension) / 10 yo	Neurosensory complications (brutal motor deficit) / 9 yo	Cutaneous sign (excision of a skin lesion for aesthetic purposes) / < 6 yo	Family screening / 11 yo	Neurosensory complications (strabismus + proptosis) / 11 yo	Family screening / ND	Family screening / 11 yo	Neurosensory complications (hearing loss) / 12 yo	Cutaneous sign (sexcision of skin lesions for aesthetic purposes at 8 and 14 yo) / <8 yo	Neurosensory complications (cervicalgy + genito-sphincter disorders) / 14 yo	Ophtalmological sign (retinal hamartoma secondarily complicated with retinal detachment) / congenital	Neurosensory complications (hearing loss + tinnitus) / 15 yo	Cutaneous signs (skin tumours) / 13 yo
**Dermatological features**	**Yes**	**Yes**	**Yes**	**Yes**	**Yes**	**Yes**	**Yes**	**Yes**	**Yes**	**Yes**	**Yes**	**Yes**	**Yes**	**Yes**	**No**	**Yes**	**Yes**	**Yes**	**Yes**	**Yes**	**Yes**
Skin tumours / Number	Yes / 3	Yes / 11	Yes / 7	Yes / 15	Yes / 3	Yes / 10	Yes / 2	Yes / 4	Yes / 12	Yes / 3	Yes / 1	Yes / 1	Yes / 7	Yes / 15	No	Yes / 8	Yes / 6	Yes / 7	Yes / 4	Yes / 2	Yes / 6
Type A / AH	1 (1 yo)	Yes (1 yo)	5 (2 yo)	2	3 (before 8 yo)	1 (after 2 yo)	0	3	10	3	0	0	7	15	0	5 (12 yo)	4 (< 8 yo)	1	0	0	5 (13 yo)
Type B	1 (congenital)	0	1	5 (10 yo)	0	1	0	0	2	0	1 (< 6 yo)	0	0	0	0	0	2	3 (< 9 yo)	4 (< 4yo)	2 (16 yo)	1 (13 yo)
Type C	1 (1 yo)	Yes (1 yo)	1 (1,5 yo)	8 (6 congenital, 2 < 6 yo)	0	8 (1 congenital, 7 > 2 yo)	2 (1 congenital)	1	0	0	0	1 (10 yo)	0	0	0	2 (12 yo)	0	3 (12 yo)	0	0	0
Type D	0	Yes (1 yo)	0	0	0	0	0	0	0	0	0	0	0	0	0	1 (12 yo)	0	0	0	0	0
Histology	Schwannoma	Plexiform Schwannoma	ND	ND	Plexiform Schwannoma	ND	ND	ND	ND	Plexiform Schwannoma	Schwannoma	ND	Schwannoma	ND	ND	Plexiform Schwannoma	1 plexiform schwannoma + 1 Neurofibroma	Schwannoma	ND	ND	Neurofibroma
CALMs	3	0	1	5 (congenital)	2	1	5	1	0	0	4	3	0	1	0	1	4	1 (congenital)	2	2 (congenital)	0
HPMs	1	0	1	0	0	1	4	3	0	0	7	0	6 (congenital)	1	0	1	0	0	3	1	5 (5 yo)
Purple lesion	0	0	0	1	0	0	1 (congenital)	0	0	0	2	0	1	0	0	0	0	0	0	0	0
Diagnosis proposed during previous consultation	No	No	No	Lipoma	No	Neurofibroma, NF1	No	No	No	No	Lipoma	No	No	No	No	No	No	No	No	No	Neurofibroma, NF1
**ophtalmological features**	**Yes**	**No**	**Yes**	**Yes**	**Yes**	**No**	**Yes**	**Yes**	**Yes**	**Yes**	**Yes**	**Yes**	**Yes**	**Yes**	**No**	**Yes**	**Yes**	**Yes**	**Yes**	**No**	**Yes**
retinal lesions	2 hamartoma (1 yo)	No	No	hamartoma (congenital)	2 epiretinal membranes	No	hamartoma (congenital)	hamartoma (congenital)	No	No	hamartoma (congenital)	No	No	hamartoma (congenital)	No	No	4 epiretinal membranes (ND)	No	hamartoma (congenital)	No	No
cataracts	No	No	Yes (< 4 yo)	Yes (6 yo)	No	No	No	No	No	Yes (4 yo)	No	No	No	0	No	No	No	No	No	No	Yes (17 yo)
strabismus	Yes (1 yo)	No	No	Yes (congenital)	No	No	Yes (8 yo)	Yes	No	No	Yes (congenital)	Yes (congenital)	Yes (1 yo)	No	No	No	No	Yes (10 yo)	No	No	No
reduced visual acuity / amblyopia	Yes / Yes (1 yo)	No / No	No / No	Yes / Yes (congenital)	No / No	No / No	Yes / No	No / No	Yes / Yes (10 yo)	No / No	Yes, myopia / No	No / No	No / No	Yes / Yes (congenital)	No / No	Yes / No	No / No	No / No	No / No	No / No	Yes / Yes (congenital)
optic meningioma	No	No	No	Yes, bilateral	Yes, unilateral	No	No	No	No	No	Yes, unilateral	No	No	No	No	No	No	No	No	No	Yes, unilateral
other	No	No	No	binocular blindness + proptosis	reduced visual field	No	N. VI palsy	No	papillary edema	No	monocular blindness	No	proptosis + N.III palsy	No	No	reduced visual field	No	No	retinal detachment + astigmatism	No	No
**neurological features**	**No**	**Yes**	**Yes**	**Yes**	**Yes**	**Yes**	**Yes**	**Yes**	**Yes**	**Yes**	**Yes**	**No**	**Yes**	**Yes**	**No**	**Yes**	**Yes**	**Yes**	**Yes**	**Yes**	**Yes**
Number of tumour	0	2	> 10	> 10	6	0	1	> 10	1	> 10	5	0	2	> 10	0	> 10	> 10	> 10	> 10	8	> 10
tumour location																					
brain		0	0	1	1		1	1	1	1	1		1	1		1	0	1	2	3	2
spine		2	> 10	> 10	4		0	> 10	0	> 10	0		1	> 10		> 10	> 10	> 10	> 10	5	8
cranial nerves (except N.VIII)		0	0	1	1		0	0	0	0	4		0	1		0	0	3	3	0	5
histological type							ND														
Schwannoma		2	> 10	> 10	0			> 10	0	> 10	3		2	> 10		> 10	> 10	> 10	> 10	4	4
Meningioma		0	0	2	5			1	1	2	2		0	2		4	0	2	3	4	3
Ependymoma		0	0	0	1			1	0	0	0		0	0		0	0	12	4	0	8
Astrocytoma		0	0	0	0			0	0	0	0		0	0		0	0	0	0	0	0
seizures	No	No	No	Yes	No	No	No	No	Yes	Yes	No	No	No	No	No	No	No	No	No	No	No
cervicalgy	No	No	No	No	No	No	No	Yes	No	No	No	No	No	No	No	No	No	Yes	No	No	No
other	No	No	No	Headache	No	Headache	No	Torticollis + headache	Intracranial hypertension	No	No	No	headache + cerebral stroke	attention deficit disorder with hyperactivity + N. XII deficit	No	headache	headache	No	No	No	No
**ENTs features**	**No**	**Yes**	**Yes**	**Yes**	**Yes**	**Yes**	**Yes**	**Yes**	**Yes**	**Yes**	**Yes**	**Yes**	**Yes**	**Yes**	**No**	**Yes**	**Yes**	**Yes**	**Yes**	**Yes**	**Yes**
Vestibular schwannoma	No	Yes / bilateral (3 yo)	Yes / unilateral (4 yo)	Yes / bilateral (6 yo)	Yes / bilateral (7 yo)	Yes / bilateral (7 yo)	Yes / bilateral (8 yo)	Yes / bilateral (9 yo)	Yes / bilateral (10 yo)	Yes / bilateral (9 yo)	Yes / bilateral (10 yo)	Yes / bilateral (11 yo)	Yes / bilateral (11 yo)	Yes / bilateral (12yo)	No	Yes/ bilateral (12 yo)	Yes / unilateral (14 yo)	Yes/ bilateral (14 yo)	Yes/ bilateral (14 yo)	Yes/ bilateral (15 yo)	Yes/ bilateral (15 yo)
Brainstem auditory evoked potentials	No	Altered bilateral (3 yo)	ND	Altered bilateral (6 yo)	Altered unilateral (7 yo)	No	No	No	No	No	No	No	ND	ND	No	ND	ND	Altered unilateral (14 yo)	Altered unilateral (14 yo)	Altered bilateral (15 yo)	Normal
hearing loss	No	No	No	Yes (6 yo)	Yes (7 yo)	No	No	No	No	No	No	No	Yes (11 yo)	Yes (12 yo)	No	Yes (12 yo)	No	No	Yes (14 yo)	Yes (15 yo)	No
vestibular syndrom	No	No	No	Yes (6 yo)	No	No	No	No	No	No	No	No	Yes (11 yo)	ND	No	No	No	No	No	Yes (15 yo)	No
peripheral facial paralysis	No	No	No	Yes (6 yo)	No	No	Yes (8 yo)	Yes (9 yo)	No	No	No	No	No	ND	No	No	No	Yes (14 yo)	No	No	Yes (15 yo)
